# Deprotective Functionalization: An Emerging Concept for Amine Reactivity

**DOI:** 10.1002/chem.202503271

**Published:** 2025-12-15

**Authors:** Irmgard Tiefenbrunner, Saad Shaaban, Nuno Maulide

**Affiliations:** ^1^ Faculty of Chemistry Institute of Organic Chemistry University of Vienna Vienna Austria

**Keywords:** atom economy, carbamates, deprotective functionalization, sulfonamides, urea

## Abstract

Protecting groups are widely employed in multi‐step syntheses with the goal to downregulate the reactivity of certain functional groups. Their use, while often unavoidable, comes with notable drawbacks including increased costs and waste, as well as typically adding two (nonstrategic) synthetic steps to a given sequence. Moreover, late‐stage deprotection of highly functionalized compounds can pose challenges. We introduced an approach termed *deprotective functionalization*, allowing for direct conversion of a protected functional group into a new one without requiring a separate deprotection event. Interestingly, in this paradigm the protecting group not only prevents side reactions, but also primes the protected functionality for selective transformations. This article provides a conceptual framework for this approach and discusses related strategies, currently limited to protected amines.

## Graphical abstract

While protecting groups are indispensable in total synthesis to avoid undesired reactivity, installation and removal of protecting groups lead to an increase in the step‐count, cost, waste production and time requirement. One approach tackling these drawbacks is deprotective functionalization, which combines deprotection and postfunctionalization in a single step, utilizing the protecting group to activate the functional group for further transformations.

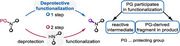

## 1 Introduction

Protecting groups (PGs) are an inescapable reality in modern target‐oriented synthesis [1, 2, 3, 4]. Although a few total syntheses have been accomplished avoiding the use of PGs [5, 6, 7, 8], most still rely on them to tame the reactivity of functional groups that would otherwise interfere with the desired reactivity at various stages of the synthetic path. Typically, the application of PGs increases the step count by two for each introduced protecting group—protection and deprotection. An efficient workaround is the use of multiple PGs that can be simultaneously cleaved under similar conditions in a global deprotection step, such as treatment with an acid [2, 4]. However, this approach becomes challenging or even impractical if the protected moieties require further functionalization during synthesis.

**SCHEME 1:**
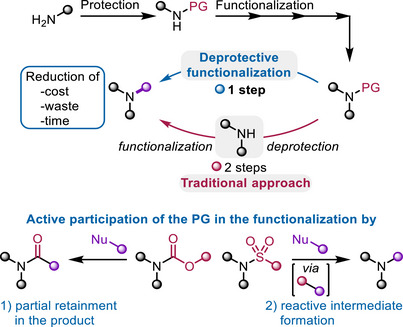
Comparison between a traditional deprotection‐functionalization approach in multistep synthesis and the novel concept of “deprotective functionalization,” the central topic of this article. PG = protecting group.

*Deprotective functionalization* is a conceptual framework that offers an alternative approach. It combines deprotection and functionalization in a single chemical step, whereby the protecting group actively participates in the functionalization event. Importantly, deprotective functionalization is strictly distinct from a “one‐pot” combination of two discrete steps (deprotection + functionalization), as it typically entails (*vide infra*) unique mechanistic pathways distinct from those involved in simple deprotection. The potential to greatly streamline reaction sequences is self‐evident (Scheme 1).

This concept article aims to gather and highlight current and scattered methods in deprotective functionalization which, to date, have been primarily confined to protected amines (particularly carbamates and electron‐poor sulfonamides).

### 1.1 Carbamates

**SCHEME 2:**
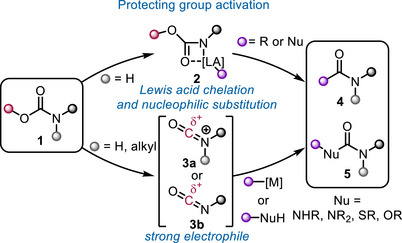
Two strategies to activate carbamates for deprotective functionalization.

Deprotective functionalization usually requires the activation of the protecting group. In the case of carbamates, this typically leads to a nucleophilic attack at the carbonyl functionality of the protecting group, either facilitated by chelation of a Lewis acid carrying the nucleophile (intermediate **2**) or through the formation of an electrophilic isocyanate **3a**/**3b**, giving access to amides, ureas, carbamates, and thiocarbamates (Scheme 2).

### 1.2 Amide Formation

**SCHEME 3:**
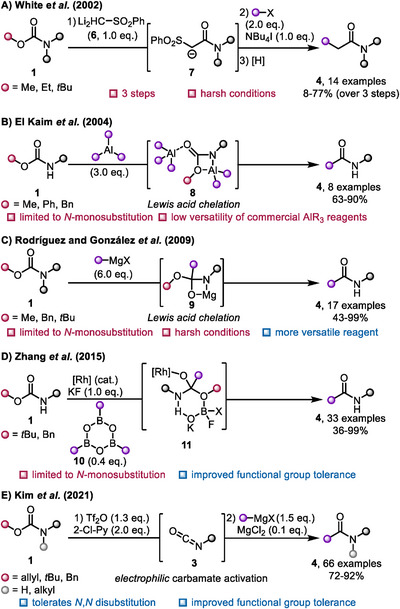
Methods for the deprotective functionalization of carbamates enabling amide formation. Black spheres represent alkyl or aryl substituents. (2‐Cl‐Py = 2‐chloropyridine, cod = 1,5‐cyclooctadiene, OTf = trifluoromethanesulfonate, Tf_2_O = trifluoromethanesulfonic anhydride, [Rh] = [Cl(cod)Rh]_2_ or [Rh(cod)_2_OTf]; **3** may be **3a** or **3b** (structures in Scheme 2).

The first deprotective amidation of carbamates was reported in 2002 by White and coworkers (Scheme 3A) [9]. Therein, they reacted carbamates with the dianion **6** of methyl phenyl sulfone, resulting in the formation of amidosulfonate **7** with alkoxide release. In order to achieve a general synthesis of amides, two subsequent steps—alkylation and reductive desulfonylation—were required. While in most cases the amidosulfone was isolated, the authors demonstrated that *in‐situ* alkylation of the amidosulfonate **7** is also feasible. Additionally, obvious limitations of the scope ensued due to the highly basic and nucleophilic nature of dianionic reagent **6**.

A more versatile approach (regarding step count) was developed by El Kaim and coworkers, who employed trialkylaluminium reagents to alkylate carbamates **1** (Scheme 3B) [10]. Here, chelation of the aluminium reagent by the oxygen and nitrogen atoms, accompanied by alkane release (leading to the formation of species **8**), were found to be crucial; the reaction did not proceed with *N*,*N*‐disubstituted carbamates.

In 2009, Rodríguez, González, and coworkers expanded this approach to allow use of structurally diverse and more readily available Grignard reagents (Scheme 3C) [11]. Although the formation of amides had been reported by several groups previously, it was typically regarded as an undesired side reaction [12, 13, 14]. In their studies, the authors reported significantly higher yields. Despite these improvements, functional group tolerance remains limited due to the harsh conditions (6 eq. of Grignard reagent) required. To prevent overalkylation to a ketone by‐product, chelation with magnesium is essential (intermediate **9**), which restricts the procedure to *N*‐monosubstituted carbamates.

A significant advancement in functional group tolerance was achieved by Zhang and coworkers, who developed an elegant rhodium‐catalyzed method that utilizes arylboroxines **10** as the aryl transfer reagent (Scheme 3D) [15]. Although the reaction still depends on monosubstituted carbamates—since coordination of the hydrogen atom to the boron reagent (species **11**) is crucial—it still shows tolerance toward reactive functional groups like esters.

Recently, Kim and coworkers introduced an alternative approach that leverages the activation of Boc‐, allyloxycarbonyl (Alloc)‐ or benzyloxycarbonyl (Cbz)‐protected amines using trifluoromethanesulfonic anhydride (Tf_2_O) in combination with a pyridine base (Scheme 3E) [16]. This leads to the formation of an isocyanate **3**, which can then be captured by a Grignard reagent. To enhance the outcome, MgCl_2_ was added to activate the Grignard reagent. Notably, esters were tolerated, highlighting the higher reactivity of the activated intermediate. Furthermore, the reaction is not limited to *N*‐monosubstituted carbamates; *N*,*N*‐disubstituted carbamates were also successfully employed.

### 1.3 Ureas, Carbamates, and Thiocarbamates

**SCHEME 4:**
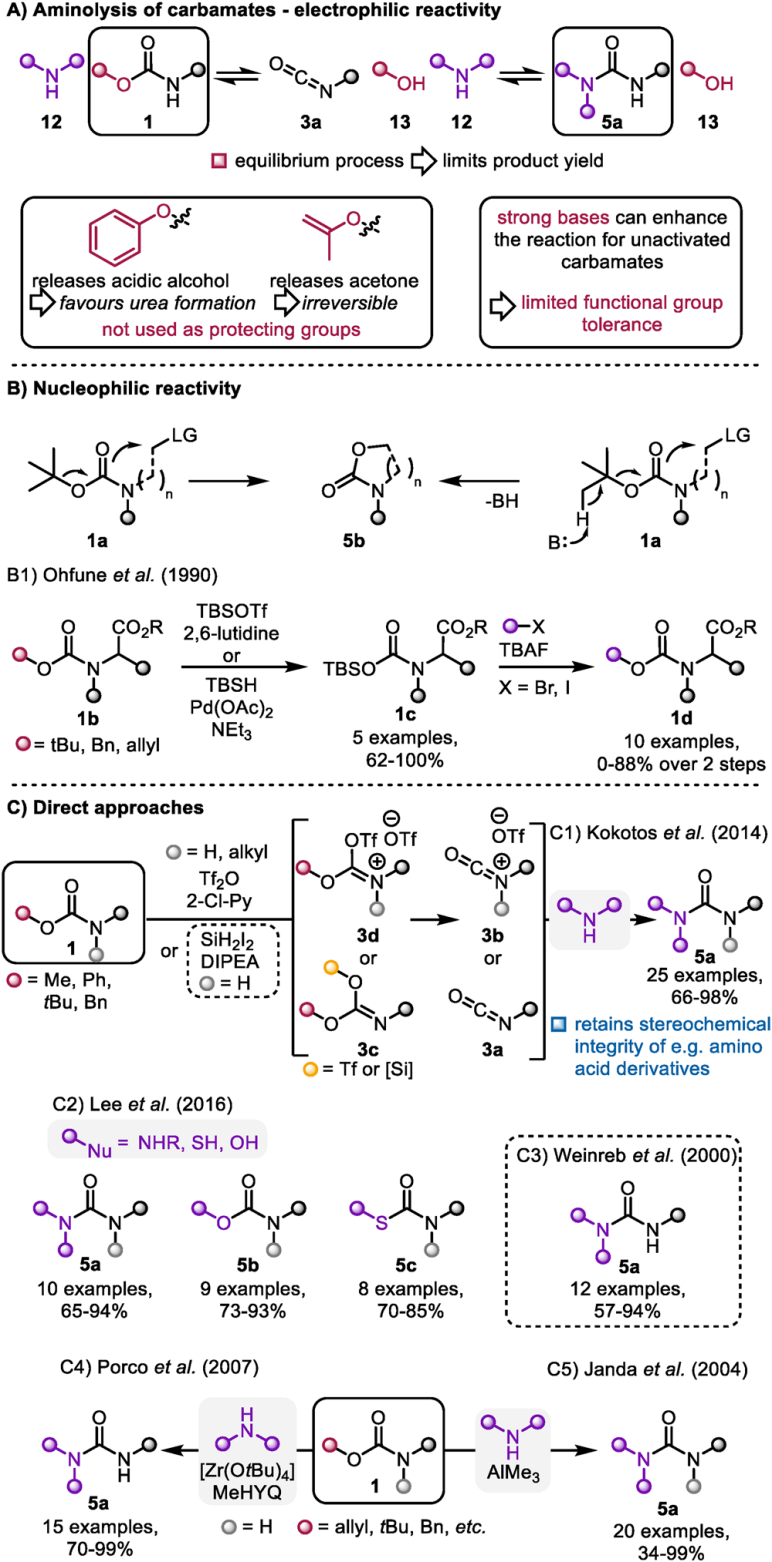
A) Aminolysis of carbamates and associated problems. B) Reactivity of carbamates (Boc) as a nucleophile. (LG = leaving group). C) Different methods for the deprotective functionalization of carbamates enabling urea, carbamate and thiocarbamate formation. Black spheres represent alkyl or aryl substituents. (DIPEA = diisopropylethylamine, MeHYQ = 4‐methylquinolin‐2‐ol, TBSOTf = *tert*‐butyldimethylsilyltrifluormethansulfonate, TBSH = *tert*‐butyldimethylsilane).

Simple aminolysis of carbamates under basic conditions or without additives has long been recognized as a viable option for urea synthesis (Scheme 4A) [17, 18, 19, 20, 21]. Typically, this is a reversible process with the equilibrium constant related to the acidity of the released alcohol [20]. To shift the reaction toward urea formation, using more acidic alcohols is advantageous, which explains why phenoxy‐substituted carbamates yield satisfactory results [17, 19]. Alternatively, Gallou and coworkers demonstrated the efficacy of isopropenyl‐substituted carbamates, where the released alcohol converts to acetone, effectively driving the equilibrium and enabling quantitative yields [20]. However, these carbamates are not commonly used as amine PGs but rather as activating agents. In contrast, when more commonly employed carbamates are subjected to aminolysis, harsher conditions, such as strong bases, are usually required [18, 21], limiting the functional group tolerance and compromising the integrity of stereochemical information. Following the same general mechanism, the synthesis of carbamates and thiocarbamates has also been reported, though again typically harsh reaction conditions are required (not shown) [22, 23, 24].

Mechanistically distinct is the reaction with an electrophile, in which the carbonyl‐oxygen (typically of a Boc group) acts as a nucleophile, expelling the *tert*‐butyl group as a carbocation or directly as isopropene if the reaction follows a concerted mechanism (Scheme 4B) [22]. While this reaction is mainly performed in an intramolecular fashion, Ohfune and coworkers have developed an approach, in which the alkyl group (*t*‐Bu, Bn or allyl) is replaced by a TBS group using TBSOTf or TBSH and a palladium catalyst (Scheme 4B1). This silyl carbamate (**1c**) can be *in‐situ* deprotected with fluoride to form an *N*‐carboxylate ion, which can react with alkyl halides to form various carbamates (**1d**) [25].

Kokotos and Lee enhanced methods for synthesizing ureas, carbamates, and thiocarbamates, which proceed *via* an isocyanate **3a** or keteniminium **3b** intermediate through reactions with amines, alcohols, or thiols, respectively. Intermediates **3a** and **3b** are presumably formed by reaction of the carbamate oxygen with Tf_2_O, forming **3c**/**3d** and subsequent elimination initiated by the attack of a nucleophile (most probably the pyridine base) at the triflyl substituent. However, the only directly observed intermediate was the isocyanate. Both groups employed similar conditions for isocyanate formation (Scheme 4, C1, and C2). Their approach utilized Boc‐ and Cbz‐protected amines and demonstrated a broad substrate scope, including esters, and alkynes, among others [21, 26].

Alternative approaches, likely proceeding *via* an isocyanate intermediate, were reported by Weinreb and Porco. Weinreb and coworkers utilized SiH_2_I_2_ to activate the carbamate (Scheme 4, C3), though *N,N*‐disubstituted carbamates were not compatible with this method [28]. Porco, on the other hand, used a Zr catalyst to convert various carbamates into ureas (Scheme 4, C4). Good functional group tolerance was demonstrated, including esters and heterocycles (indoles) [29].

Janda and coworkers developed a trimethylaluminium‐mediated urea formation, which successfully accommodated both *N*‐monosubstituted and *N*,*N*‐disubstituted carbamates (Scheme 4, C5). However, functional group tolerance was largely limited [30].

### 1.4 Sulfonamides

Deprotective functionalization of carbamates is restricted to interchanging various carbonyl‐based functional groups, often proceeding in a mechanistically relatively straightforward way. Achieving the same functionalization from a sulfonamide—namely exchange of the sulfonyl with a carbonyl group—is more challenging, requiring mechanistically more involved strategies.

**SCHEME 5:**
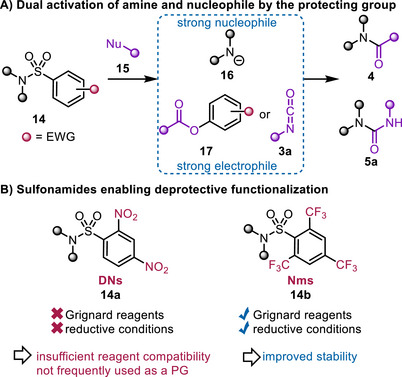
A) Concept of deprotective functionalization of sulfonamides. B) Sulfonamides successfully applied in deprotective functionalization.

Several research groups have investigated such approaches in recent decades, demonstrating that electron‐deficient aromatic sulfonamides are also amenable to deprotective functionalization [31, 32, 33, 34, 35]. Here, an external nucleophile **15** attacks the sulfonamide, releasing the amide **16**, a highly potent nucleophile (Scheme 5A). Hence, the protecting group plays a dual role, not only activating the former nucleophile **15** by converting it into an electrophilic species **17** or **3a** but also facilitating nucleophilic attack. This dual activation enables the formation of amide **4** or urea **5a**.

As the external nucleophile needs to be capable of attacking the sulfonyl protecting group, only sulfonamides with multiple electron‐withdrawing groups have been successfully employed in these deprotective functionalization approaches. The most notable examples are nonafluoromethylbenzenesulfonamide (Nms) [33, 34] and 2,4‐dinitrobenzenesulfonamide (DNs) [31, 32] (Scheme 5B).

First applied in deprotective functionalization in 1998 by Tomkinson and coworkers [31, 32], DNs exhibited the drawback of extreme lability due to strong electron deficiency and the presence of reactive nitro groups, limiting its usefulness as a protecting group [36]. In 2023, Maulide and coworkers developed the Nms protecting group [36], which addressed limitations such as the incompatibility with Grignard reagents and reductive conditions. This improvement was achieved by applying nonreactive CF_3_ groups as the electron‐withdrawing moieties. Both groups developed approaches for the conversion of sulfonamides into amides and ureas, alongside related functionalities.

### 1.5 Amides and Thioamides

**SCHEME 6:**
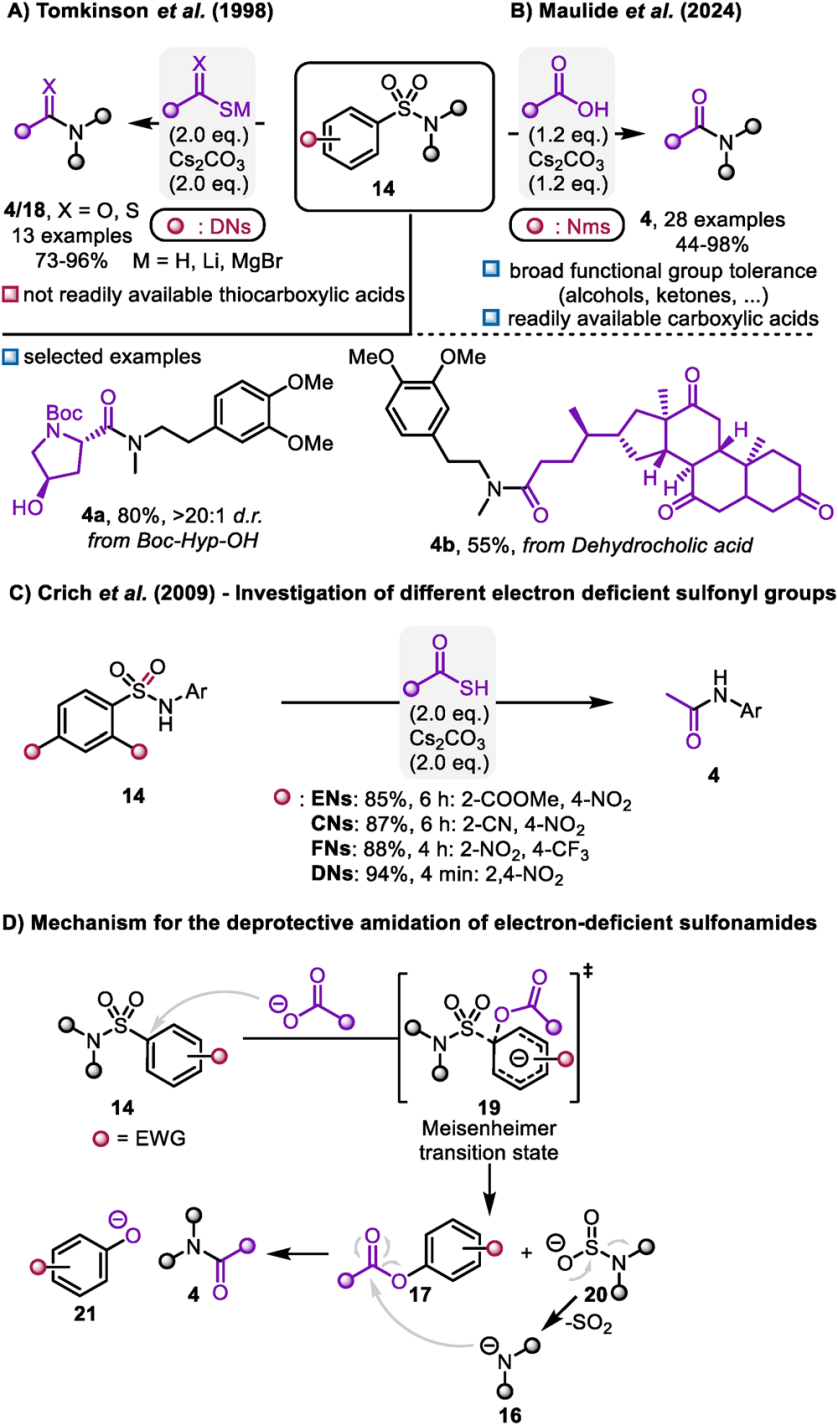
A‐C) Methods for deprotective functionalization of sulfonamides enabling amide and thioamide formation. D) Representative mechanism for the deprotective functionalization of sulfonamides with carboxylates as nucleophiles.

While Tomkinson's method for accessing amides (**4**) and thioamides (**18**) was limited to the use of thiocarboxylic acids and dithioacid nucleophiles (Scheme 6A) [31, 32], Maulide expanded the scope to include the much more accessible carboxylic acids and reduced the required amount of acid (Scheme 6B) [33]. Utilizing less reactive nucleophiles and a less reactive sulfonamide necessitated a higher temperature. Due to the ready availability of the coupling partners (carboxylic acids), a broad functional group tolerance was demonstrated, accommodating unprotected alcohols (**4a**), ketones (**4b**), secondary amides, nitriles, and Boc‐protected amines further highlighting the advantage of this method.

The application of deprotective functionalization in peptide synthesis offers a valuable approach for constructing peptides. Crich and coworkers explored the use of various sulfonamides for peptide synthesis (not including Nms) [35, 37]. To this end, they compared the reactivity of different electron‐deficient sulfonamides with thioacetic acid. DNs proved to be the most reactive, completing the reaction within a few minutes, whereas other sulfonamides (ENs, CNs, and FNs, see Scheme 6C) required several hours. This reactivity allows for orthogonal coupling of DNs‐protected peptides in the presence of other PGs. Hence, two consecutive coupling steps are possible. Furthermore, compatibility with thioesters was demonstrated making this method suitable for subsequent native chemical ligation.

The mechanism relies on the electron deficiency of the sulfonamide **14**, enabling a nucleophilic attack at the *ipso* position of the aromatic ring (Scheme 6D). Computational studies performed by the Maulide group (for a carboxylate nucleophile) [33] indicated that the reaction proceeds via a concerted mechanism, with a simultaneous C‐O bond formation and C‐S bond cleavage occurring through a Meisenheimer transition state **19**. The resulting amidosulfinate **20** then undergoes SO_2_ extrusion, releasing amide **16**. Subsequently, this can attack the liberated activated ester **17**, generating the desired amide **4** along with a phenolate **21**.

**SCHEME 7:**
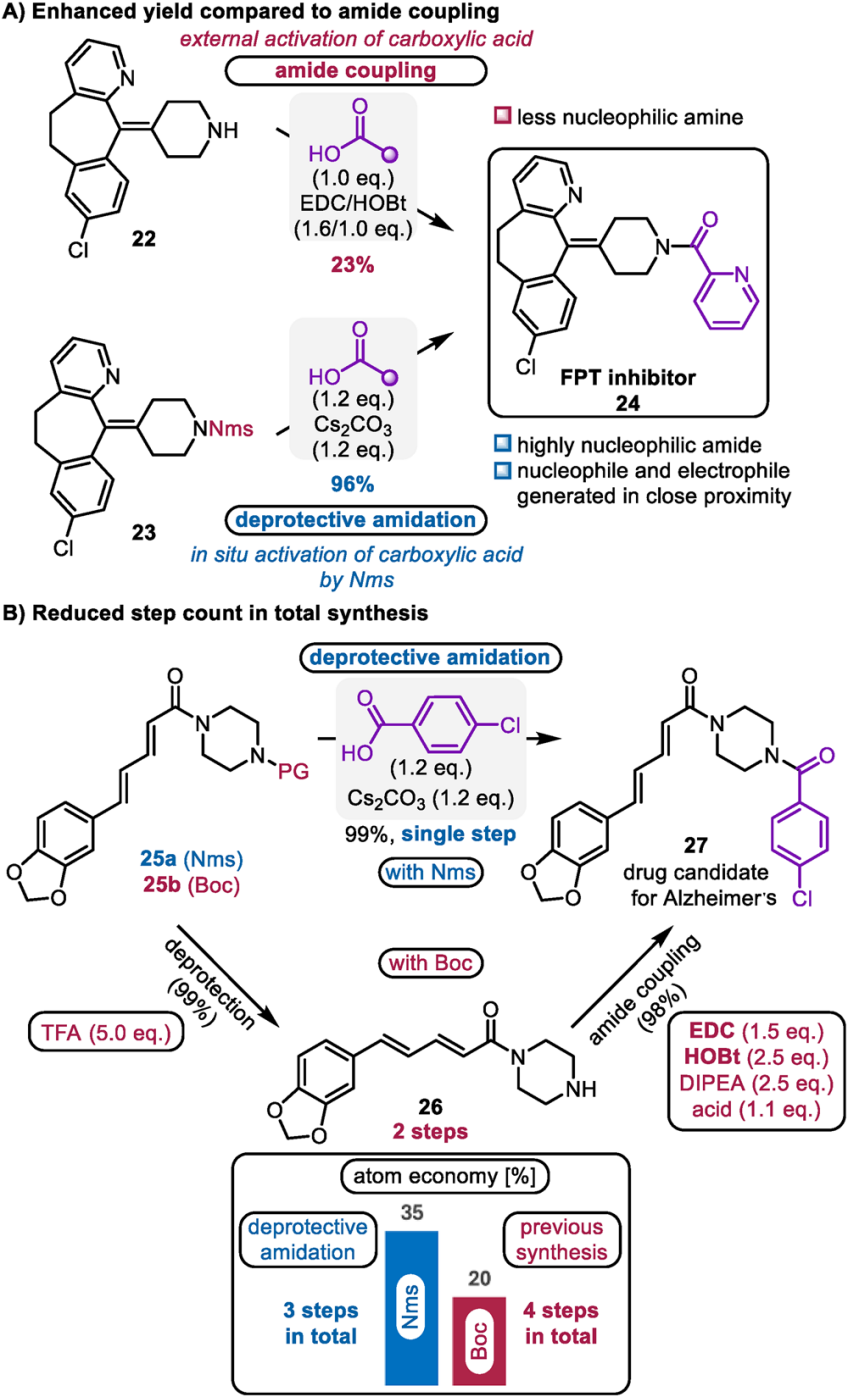
A) Case study showing the benefit of deprotective amidation over amide coupling. (FPT = farnesyl protein transferase). B) Application of deprotective amidation in the total synthesis of a drug candidate. (EDC = 1‐ethyl‐3‐(3‐dimethylaminopropyl)carbodiimide, HOBt = hydroxybenzotriazole, TFA = trifluoroacetic acid).

The value of the deprotective functionalization approach is not exhausted in its improvements to step count. The Maulide group demonstrated that using deprotective functionalization of Nms amides can substantially improve the obtained yield of various farnesyl protein transferase (FPT) inhibitors (**24**) compared to conventional amide coupling with EDC/HOBt (Scheme 7A) [33]. The key difference between these reactions is that for classical amide coupling an external reagent triggers the activation process. In deprotective functionalization, the protecting group itself serves as the activator. This mechanism generates a highly nucleophilic amide in close proximity to the *in situ* formed electrophile: this proximity, combined with the stronger nucleophilic character of amides, are likely reasons for the improved efficiencies afforded by deprotective functionalization.

Undeniably, deprotective functionalization shortens synthetic sequences (the path toward drug candidate **27** goes from 4 to 3 steps by eliminating the need for a separate deprotection and subsequent amide coupling, cf. Scheme 7B). This also improves atom economy by avoiding the use of coupling reagents such as EDC and HOBt [38].

### 1.6 Ureas

**SCHEME 8:**
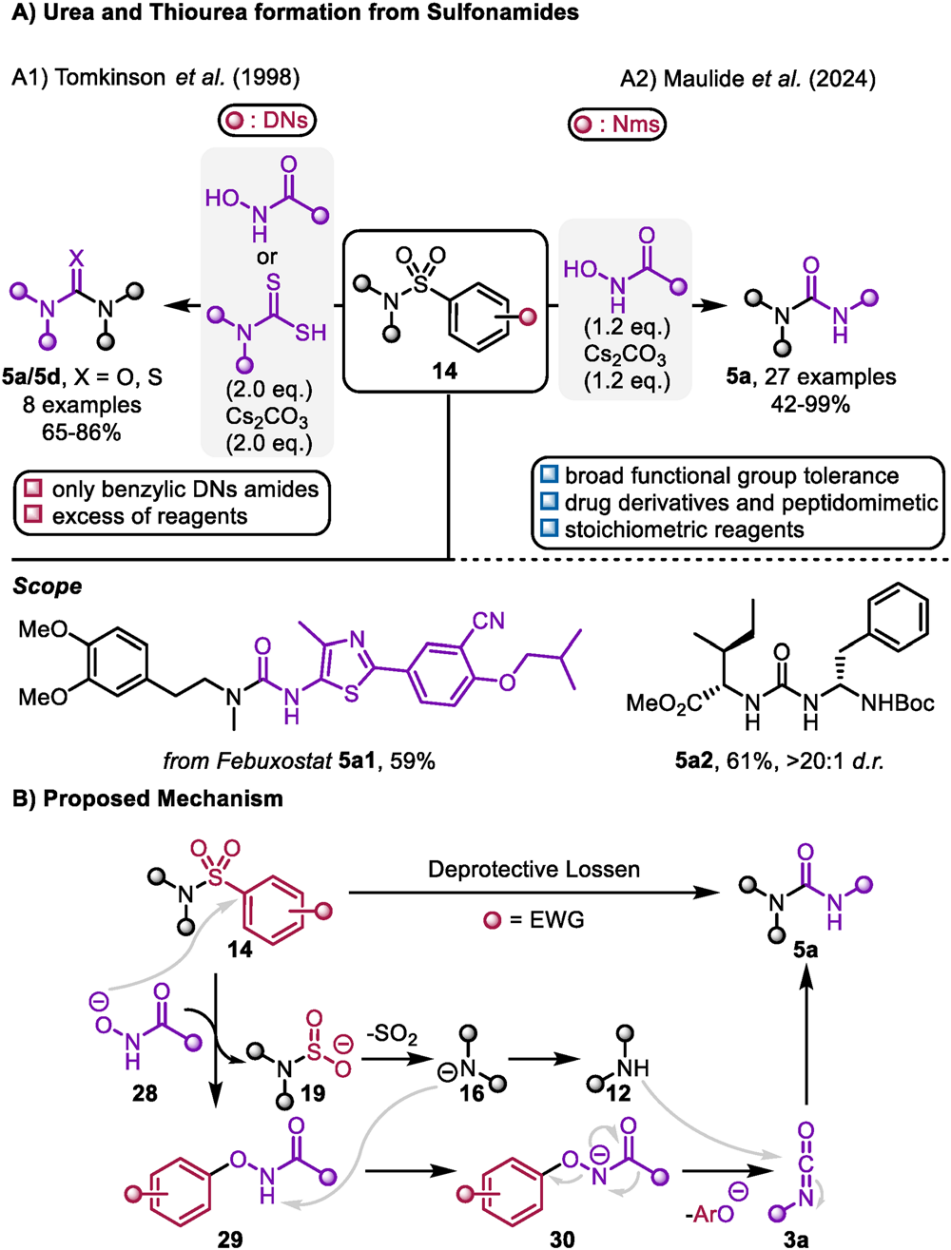
A) Methods for the deprotective functionalization of sulfonamides enabling urea and thiourea formation. B) Proposed mechanism of the deprotective functionalization of sulfonamides to access urea derivatives. Black spheres represent alkyl or aryl substituents.

Tomkinson demonstrated that DNs‐amides (**14a**) serve as versatile starting materials not only for amide and thioamide synthesis but also for accessing ureas (**5a**) and thioureas (**5d**) when treated with hydroxamic acids or dithioamides, respectively (Scheme 8A1) [32]. In the case of urea, the reaction seemingly proceeds through an acyl(aryloxy)amide intermediate (**30**), which rearranges into an isocyanate (**3a**). This intermediate is crucial, as *N*,*N*‐disubstituted hydroxamic acids fail to produce the desired urea, resulting in amine deprotection. Furthermore, Tomkinson reported similarities of the accessible scope with the Lossen rearrangement, with certain substituents, particularly primary alkyl groups like methyl, exhibiting low migration tendencies and ultimately leading to amine deprotection rather than urea formation.

While interesting from a conceptual point of view, the utility of this transformation was again somewhat hampered by the low stability of DNs. Recently, Maulide extended this chemistry to Nms‐amides (Scheme 8A2). Unlike in the studies undertaken by Tomkinson and coworkers, a broad functional group tolerance, including unprotected alcohols, esters, nitriles, indoles, and ketones was demonstrated. Additionally, several amine‐containing drugs (**5a1**) were successfully functionalized and a peptidomimetic (**5a2**) was synthesized with this approach. The reduction in reagent quantities required enhances the appeal of this method, particularly for large‐scale application [34]. Moreover, the increased stability of Nms compared to DNs makes late stage applications (after usage as a protecting group) more feasible [33].

Building on the observations made by Tomkinson et al. and Maulide et al., the following mechanism for this transformation is proposed: the hydroxamic acid (**28**) attacks the electrophilic *ipso* position of the protecting group, leading to the release of amidosulfinate **19**. Upon extrusion of SO_2_, amide **16** can deprotonate intermediate **29**, followed by release of the phenolate and 1,2‐migration of the substituent, forming isocyanate **3a**, either with intermediacy of a nitrene or in a concerted manner. **3a** can then react with the amine **12** to yield the desired urea **5a** (Scheme 8B) [34].

### 1.7 Isoxazolidines

**SCHEME 9:**
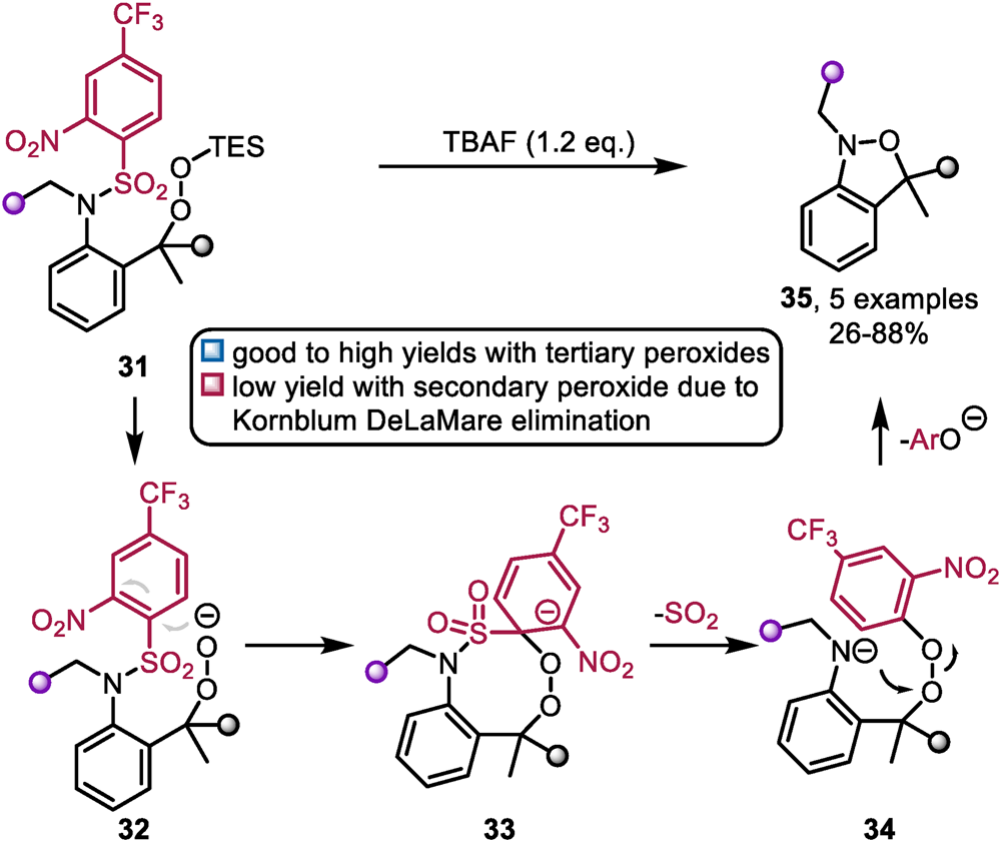
Mechanism of the isoxazolidine synthesis developed by Crich and coworkers (2025).

Recently Crich and coworkers utilized the deprotective functionalization concept for the synthesis of isoxazolidines from 3‐(4‐trifluoromethyl‐2‐nitrobenzenesulfonamido)alkyl silylperoxides in an intramolecular cyclization event under *N*‐*O* bond formation [39]. The reaction is initiated by peroxide deprotection with tetrabutylammonium fluoride (TBAF). The thus formed peroxy anion (**32**) attacks the electron poor sulfonyl protecting group, which, under extrusion of SO_2_, leads to the release of the amide (**34**) in close proximity to the newly formed aryl peroxide—a highly reactive scaffold. A subsequent nucleophilic attack of the amide on the peroxide, resulting in phenolate release, gives the desired isoxazolidine product (**35**). This reaction proceeds readily with high yields when tertiary peroxides are used. However, yields significantly drop when employing less sterically hindered secondary peroxides, as the Kornblum‐DeLaMare elimination becomes a competitive reaction pathway (Scheme 9).

This example, alongside the deprotective Lossen reaction in particular illustrate the appeal of the deprotective functionalization approach. Indeed, by careful design of the protecting group it becomes useful to tame the functional group's reactivity, but also serves as a reactivity handle. Upon reaction with a nucleophile, two highly reactive intermediates (or one containing both the nucleophilic as well as the electrophilic reaction partners) are transiently formed, allowing for productive downstream reactivity. In this way, the protecting group fulfills multiple crucial roles.

### 1.8 Sulfonyl Migration

**SCHEME 10:**
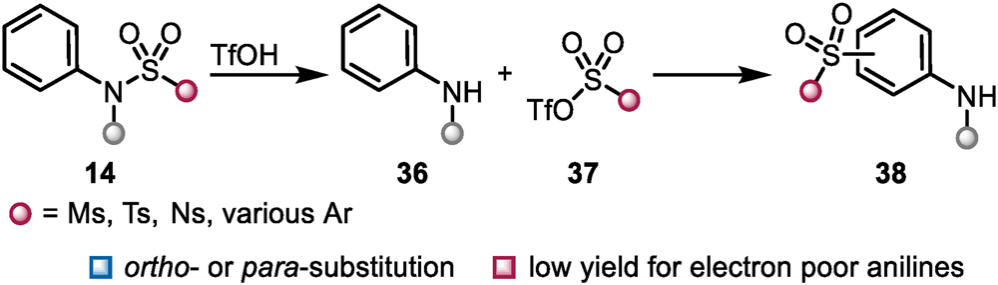
Acid catalyzed Fries‐type rearrangement of sulfonyl‐substituted anilines.

A distinct mode of reactivity—the acid‐catalyzed sulfonyl migration—was discovered by Witt and Uermenyi more than a century ago and was further developed by Zanger, Orentas, and others [40, 41, 42]. The reaction is initiated by the protonation of the sulfonamide at the nitrogen, followed by *N*‐*S* bond cleavage under release of aniline **36** and activated species **37** (Scheme 10). Subsequently, an electrophilic aromatic substitution occurs, leading to *ortho*‐ or *para*‐substituted anilines (**38**). While other strong acids (H_2_SO_4_, PPA) sometimes also trigger this Fries‐type rearrangement, TfOH proved to be more reliable, particularly when *N*‐unsubstituted anilines were used, which often only undergo deprotection, but no subsequent sulfonyl incorporation [41]. While initially harsh conditions were used (acid as a solvent), Orentas reduced the amount of required acid to only 2.0 eq., making the process milder and more environmentally friendly [42].

### 1.9 Summary and Outlook

While PGs are essential to most total syntheses, they come with several drawbacks, including reduced atom‐economy, increased costs, and reduced time efficiency. In certain cases, when postfunctionalization after deprotection is desired, these challenges can be partially circumvented by employing a deprotective functionalization strategy—meaning that deprotection and functionalization proceed in a single step with the protecting group actively participating in the functionalization. Although first approaches based on deprotective functionalization logic sporadically appeared throughout of the 20^th^ century and recent years have witnessed significant advancements in this field, the concept as such was only established in 2024 by Maulide and coworkers. Currently, methods enabling this strategy are restricted to carbamates and foremost electron‐poor sulfonamides. As such, substantial potential for further growth and exploration remains. We hope that establishing this concept will inspire the community and we expect new discoveries in the field of deprotective functionalization to be made.

## Conflicts of Interest

The authors declare no conflict of interest.
